# Outcome of children with life-threatening asthma necessitating pediatric intensive care

**DOI:** 10.1186/1824-7288-36-47

**Published:** 2010-07-06

**Authors:** Kam-Lun Hon, Wing-Sum Winnie Tang, Ting-Fan Leung, Kam-Lau Cheung, Pak-Cheung Ng

**Affiliations:** 1 Department of Pediatrics, The Chinese University of Hong Kong, Prince of Wales Hospital, Hong Kong; 2 Department of Family Medicine, University at Buffalo, Erie County Medical Center, 462 Grider St. Buffalo NY, 14215 USA

## Abstract

**Objective:**

To report the outcome of children with life-threatening asthma (LTA) admitted to a university Pediatric Intensive Care Unit (PICU).

**Methods:**

Retrospective study between October 2002 and May 2010 was carried out. Every child with LTA and bronchospasm was included.

**Results:**

30 admissions of 28 patients (13 M, 17 F) were identified which accounted for 3% of total PICU admissions (n = 1033) over the study period. The majority of patients were toddlers (median age 3.1 years). Few had past history of prematurity, lung diseases, or neuro-developmental conditions. Approximately half had previous admissions for asthma and one-forth with history of non-compliance to recommended treatment for asthma. One patient had parainfluenza virus and one had rhinovirus isolated. None of these factors were associated with need for mechanical ventilation (n = 6 admissions). Comparing with patients who did not receive mechanical ventilation, ventilated children had significantly higher PIM2 score (1.65 versus 0.4, p < 0.001), higher PCO_2 _levels (9.3 kPa versus 5.1 kPa, p = 0.01) and longer PICU stay (median 2.5 days versus 2 days, *p *= 0.03) The majority of patients received systemic corticosteroids, intravenous or inhaled bronchodilators. There was one pneumothorax but no death in this series.

**Conclusions:**

LTA accounted for a small percentage of PICU admissions. Previous hospital admissions for asthma and history of non-compliance were common. Approximately one quarters required ventilatory supports. Regardless of the need for mechanical ventilation, all patients survived with prompt treatment.

## Introduction

Asthma is a very common childhood condition worldwide and in Hong Kong. Acute asthmatic attacks cause significant morbidity and account for a significant number of emergency department consultations and hospital admissions[1-4]. Most children admitted to the hospital because of acute asthma do not require intensive care treatment. Nevertheless, a small percentage of children with life-threatening asthma (LTA) would develop progressive respiratory failure refractory to treatment and require admission to the pediatric intensive care unit (PICU)[5-7]. In those who are admitted to the ICU, approximately 10 to 33% need intubation and mechanical ventilation, with a risk of worsening bronchospasm and hyperinflation, barotrauma, and cardiovascular depression[8,9]. If not promptly managed, severe asthmatic attacks may occasionally result in death[1-10]. The purpose of this study was to report the clinical pattern and outcome of all children with LTA and severe bronchospasm admitted to the PICU.

## Methods

We retrospectively reviewed the medical records and analyzed data from all children with LTA admitted to the PICU of a tertiary care university hospital (Prince of Wales Hospital) in Hong Kong during the period October 2002 and May 2010. LTA was defined as all children with asthma who required ICU admission and care. The initial diagnosis was made clinically by the admitting physicians. Final diagnosis was confirmed on chart review and subsequent evaluations. The hospital provides PICU care to a catchment population of approximately 1.1 million. The following data were collected: age, sex, duration of admission, treatment of the LTA, clinical condition, blood gases, the incidence of barotrauma, and outcome. Respiratory viruses and bacterial pathogens were routinely screened for by standard examination of nasopharyngeal aspirate and cultures. The Pediatric Index of Mortality 2 (PIM2) score based on admission data was used as severity score[11]. Numerical data were compared with Mann Whitney U test and categorical data with χ^2 ^or Fisher exact test. All comparisons were made two-tailed, and p-values less than 0.05 considered statistically significant.

## Results

There were 30 admissions (13 boys and 17 girls; median age, 3.1 years; IQR 2.0 - 6.8 years; Table 1) with LTA which accounted for 3% of total PICU admissions (n = 1033) over a study period of 7 years and 8 months. Two male patients were admitted twice because of a recurrent episode of LTA. Indications for admission to the PICU were severe dyspnea, worsening or failure to improve on nebulized bronchodilators, and need for administration of intravenous salbutamol or mechanical ventilation. The decision for PICU admission was determined clinically together with blood gas as well as pulse oximetry parameters by the admitting physicians.

**Table 1:** Clinical data of children with status asthmaticus admitted to PICU

Case	Total (n = 30)	Ventilated (n = 6)	Non-ventilated (n = 24)	P*
Male (%)	13 (0)	5 (75)	8 (40)	0.06
Median age (IQR), yr	3.1 (2.0-5.4)	3.3 (2.0-5.5)	3.1 (1.9-6.8)	0.84
Median (IQR) PIM2, %	0.50 (0.30-1.00)	1.65 (1.45-1.95)	0.4 (0.3-0.5)	< 0.001
**Relevant risk factors**				
Family history of atopy	12	1	11	0.36
Prematurity < 36 weeks (%)	4 (14)	0 (12)	4 (15)	0.57
History of chronic lung disease, bronchiolitis, pneumonia, or pneumothorax (%)	7 (17)	0 (0)	7 (25)	0.29
Neurodevelopmental delay (mental retardation, cerebral palsy, neuromuscular disease) (%)	2 (14)	0 (25)	2 (10)	1.00
Previous asthma admission (%)	16 (50)	4 (62)	12 (45)	0.66
Maintenance inhaled CS (%)	12 (35)	3 (37)	9 (35)	0.66
Non-compliance (%)	7 (25)	2 (25)	5 (25)	0.60
1^st ^PaCO_2 _in PICU, kPa	5.5 (4.3-7.9)	9.3 (6.4-10.9)	5.1 (4.3-5.9)	0.01
1^st ^PaO_2 _in PICU, kPa	9.9 (7.8-13.2)	10.1 (9.4-13.5)	9.3 (7.5-13.3)	0.40
Viral isolation (%)	2 (3)	0 (0)	2 (5)	1.00
**Treatment at PICU**				
Systemic CS (%)	30 (100)	6 (100)	24 (100)	N/A
Inhaled salbutamol (%)	22 (73)	5 (83)	17 (71)	1.00
Inhaled ipratropium (%)	7 (23)	0 (0)	7 (29)	0.29
Inhaled adrenaline (%)	2 (7)	1 (17)	1 (4)	0.37
Intravenous salbutamol (%)	22 (73)	5 (83)	17 (71)	1.00
Intravenous magnesium sulphate (%)	4 (13)	2 (33)	2 (8)	0.17
Systemic antibiotics (%)	19 (63)	5 (83)	14 (58)	0.37
**Outcome**				
Median (IQR) PICU stay, day	2.0 (1.0-2.5)	2.5 (2.0-4.5)	2.0 (1.0-2.0)	0.03
Median (IQR) hospital stay, day	5.0 (3.0-7.5)	6.5 (3.0-20.0)	5.0 (3.0-6.8)	0.27
Pneumothorax (%)	1 (3)	0 (0)	1 (4)	1.00
Died in PICU	0	0	0	N/A

CS: corticosteroid; IQR, interquartile range; N/A, not applicable.

* Analyzed between ventilated and non-ventilated patients by Fisher exact test or Pearson χ^2 ^for categorical variables and Mann-Whitney U test for numerical variables.

In terms of risk factors, 'smoker(s) at home' were present in 5 of the admissions, 'history of atopy in 1 st degree relative' in 11 admissions, and 'personal history of atopy' in 20 admissions. Few had past history of prematurity (n = 4 admissions), lung diseases (1 neonatal pneumothorax, 1 pneumonia, 1 chronic lung disease, and 4 recurrent bronchiolitis), neuro-developmental condition (Rasmussen's encephalitis plus epilepsy × 2 admissions). Half had previous admissions for asthma and one-fourth with history of non-compliance to recommended treatment for asthma. In the ventilated group, three patients were on inhaled corticosteroid but compliance to corticosteroid was reportedly poor in two. In the non-ventilated group, 9 patients were on inhaled corticosteroid. One patient also received oral montelukast. Poor-compliance to asthma management was reported in 5 patients. One patient had parainfluenza virus and one had rhinovirus isolated. None of these factors were associated with need for mechanical ventilation which was required in 6 patients. Ventilator modes included Synchronized Intermittent Mandatory Ventilation (SIMV) or Pressure Regulated Volume Control mode (PRVC), with low Positive End-Expiratory Pressure (PEEP) and low inspiratory to expiratory (I:E) ratios. Comparing with patients who did not receive mechanical ventilation, ventilated children had significantly higher PIM2 score (1.65 versus 0.4, p < 0.001), higher first PaCO_2 _levels measured at PICU (9.3 kPa versus 5.1 kpa, p = 0.01) and longer PICU stay (median 2.5 days versus 2 days, *p *= 0.03) but no differences in other factors evaluated in Table 1. These patients received systemic corticosteroids and intravenous or inhaled bronchodilators. Some received intravenous magnesium sulphate. There was one pneumothorax but no death in this series. A 7-year-old girl with asthma but no previous asthma hospitalization, presented with sudden dyspnoea following a 2-day history of blocked nose and cough. Her private practitioner prescribed inhaled and oral bronchodilators. However, dyspnoea was not relieved and chest radiograph at the emergency department revealed left apical pneumothorax, which was drained with a chest drain. CT scan of the thorax showed subcutaneous emphysema, pneumomediastinum, and pneumothorax. Her symptoms resolved at the PICU following corticosteroid and inhaled salbutamol. Mechanical ventilation was not required. The chest drain was removed three days later. Regardless of mechanical ventilation, all had very brief PICU stays (median 2 days; range, 1 to 7 days). Furthermore, there did not appear to be any increase in incidence of PICU admissions for LTA between 2003-2009 (median 2% of PICU admissions; Table 2).

**Table 2:** The incidence of PICU admissions for LTA during the study period

Year	PICU admissions	PICU for LTA (%)
2002 (partial data)	33	1 (3)
2003	122	7 (6)
2004	155	3 (2)
2005	111	2 (2)
2006	127	3 (2)
2007	144	5 (3)
2008	138	3 (2)
2009	140	3 (2)
2010 (partial data)	63	3 (5)

Median of 2% of annual PICU admissions were due to LTA (average absolute deviation from Median = 1.0%)

## Discussion

### Incidence of PICU admissions

Asthma is a common disease and its frequency of occurrence sometimes detracts from its potential seriousness[12]. Severe asthma in children is a frequent cause of hospital and pediatric ICU admissions in reported series[12-14]. Globally, morbidity and mortality associated with asthma have increased over the last 2 decades[1,2,4]. This increase is attributed to increasing urbanization and undertreatment of asthma especially among the high risk pediatric population with low-socio-economic class[15]. Despite advancements in our understanding of asthma and the development of new therapeutic strategies, the morbidity and mortality rates due to asthma reportedly increased between 1980 and 1995[2,4,13]. In the United States, the mortality rate due to asthma has increased in all age, race, and sex strata. From 1975-1993, the number of deaths nearly doubled in people aged 5-14 years[2]. In Hong Kong, data about severe asthma hospitalizations are lacking. In a previous study, we reported that asthma accounts for approximately 10% of general pediatric admissions[3]. In the present study, the admission rate was only 3% of PICU admissions. It appears that LTA is a relative uncommon cause of PICU admission in our locality. The reason for this is unknown. It might reflect that treatment received at the emergency department is prompt and effective to halt PICU admission[6].

### PICU treatment

Status asthmaticus is severe asthma that does not respond well to immediate care and is a life-threatening medical emergency[5]. Ensuing respiratory failure results in hypoxia, carbon dioxide retention and acidosis[16]. Patients require aggressive treatment with oxygen, bronchodilators, and corticosteroids[5,17-19]. Rapid reversal of airflow obstruction is achieved by using repeated or continuous administration of an inhaled beta2-agonist. Early administration of systemic corticosteroids (oral or intravenous) is indicated in children with LTA[5,19]. In severe cases, alveolar hypoventilation requires mechanically assisted ventilation[1,5,8,16]. In our study, the only significant difference between ventilated and non-ventilated group is the presence of CO_2 _retention. LTA can be associated with metabolic acidosis, which reduces the effectiveness of ß-agonists[9]. In a prospective randomized trial, continuous nebulization of albuterol is safe and results in more rapid clinical improvement than intermittent nebulization in children with impending respiratory failure due to status asthmaticus[20]. In severe LTA attacks, intravenous salbutamol was found to be a safe and effective bronchodilator capable of reversing severe bronchospasm in most children who would otherwise require mechanical ventilation[21]. Mechanical ventilation compromises active expiration with increased air trapping and hypercapnia, and should therefore be delayed as long as possible by using medical therapy[1]. Noninvasive positive pressure has also been advocated but further evaluation of its efficacy is required[22].

### Co-infections

One of the clinical problems facing pediatric intensivists is the differentiation between viral and bacterial infections when an acutely ill child with LTA is admitted. Empirical course of antibiotics were often used in the initial management in order to avoid missing any treatable bacterial co-infections. Rapid diagnosis of respiratory viral infections in children is important because prompt diagnosis may result in significantly reduced antibiotic use and unnecessary strict isolation for respiratory viruses. In this study, there was no bacterial isolation and viral co-infection was only found in two cases (parainfluenza and rhinovirus).

### Complications and morbidity

Status asthmaticus is one of the most common causes of admission to a pediatric intensive care unit (PICU). There have been published data examining the complications associated with the treatment of status asthmaticus in children in the PICU. In one study of children admitted to PICU, there was a 22% complication rate, increased by intubation[14]. The risk of death is increased where there is delay in getting treatment, particularly time to starting steroids. Another retrospective review showed 8% of children admitted to the ICU with status asthmaticus experienced one or more complications during their treatment. The most common complications were aspiration pneumonia, ventilator-associated pneumonia, pneumomediastinum, pneumothorax, and rhabdomyolysis[23]. Intubated children were significantly more likely than non-intubated children to experience a complication (RR 15.3; 95% CI 6.7-35). Intubated children experiencing a complication also had significantly longer duration of mechanical ventilation, ICU length of stay, and hospital charges than intubated children not experiencing a complication, suggesting that intubation and mechanical ventilation itself may increase the risk of developing a complication in this population[7,14]. Asthma patients have variable resolution of airway obstruction during mechanical ventilation and controlled hypoventilation can be a safe therapy for the patients with more severe obstruction[8]. Prompt treatment with corticosteroid, bronchodilator (intravenous route if needed), magnesium sulphate, permissive hypercarbia, and the avoidance of mechanic ventilation together might have accounted for the satisfactory outcome seen in our patients. Indeed mechanical ventilation has not been needed for the past 3 years in our unit.

Near-fatal asthma continues to be a significant problem despite the decline in overall asthma mortality. Two distinctive phenotypes of near-fatal asthma have been identified: one with eosinophilic inflammation associated with a gradual onset and a slow response to therapy and a second phenotype with neutrophilic inflammation that has a rapid onset and rapid response to therapy. In stable condition, near-fatal asthma frequently cannot be distinguished from mild asthma. Diminished perception of dyspnea plays a relevant role in treatment delay, near-fatal events, and death in patients with severe asthma. Reduced compliance with anti-inflammatory therapy has also been associated with fatal or near-fatal asthma[24]. The sudden-onset patients were older and they more commonly presented to the emergency department between midnight and 8:00 am with severe exacerbations that required intubation and intensive care unit admission. Nevertheless, this sudden-onset group was discharged from the hospital earlier[25]. The preventable factors included inadequate assessment or therapy of prior asthma, poor compliance with therapy, and delay in seeking help[10]. In our series, history of previous asthma admissions and noncompliance were also relevant factors. Nevertheless, we report no fatality and the majority required brief PICU stay regardless of need for mechanical ventilation.

Approximately one third of patients were on maintenance inhaled corticosteroid; many of these patients were on inhaled salbutamol on a *prn-*basis and had been asymptomatic for a number of months, but some were non-compliant to prescribed therapy. The limitation of this study is the small sample size and there was high chance of type II error with regard to different parameters studied.

In conclusion, LTA and bronchospasm accounted for a small percentage of PICU admissions following initial stabilization at the emergency department. Approximately 20% required ventilatory supports. Ventilated patients appeared to have higher PIM2 severity score, higher PCO_2 _and longer PICU stays. Regardless of the need for mechanical ventilation, all patients survived with prompt treatment, and only required brief PICU stays.

## Competing interests

The authors declare that they have no competing interests.

## Authors' contributions

KLH is the principal author, WSWT is involved with data review, TFL provides statistical assistance, KLC is involved in patient care and provides inputs and audit for the manuscript, PCN is involved in review of the manuscript. All authors read and approved the final manuscript.

## References

[B1] Werner HA Status Asthmaticus in Children: A Review Chest 2001 119 1913 1929 10.1378/chest.119.6.1913 11399724

[B2] Akinbami LJ Schoendorf KC Trends in childhood asthma: prevalence, health care utilization, and mortality Pediatrics 2002 110 2 Pt 1 315 322 10.1542/peds.110.2.315 12165584

[B3] Hon KL Nelson EA Gender disparity in paediatric hospital admissions Ann Acad Med Singap 2006 4 2 882 888 17219000

[B4] Moorman JE Rudd RA Johnson CA King M Minor P Bailey C National surveillance for asthma--United States, 1980-2004 Morbidity & Mortality Weekly Report 2007 56 1 54 17947969

[B5] Stein R Canny GJ Bohn DJ Reisman JJ Levison H Severe acute asthma in a pediatric intensive care unit: six years' experience Pediatrics 1989 83 1023 1028 2726328

[B6] Pirie J Cox P Johnson D Schuh S Changes in treatment and outcomes of children receiving care in the intensive care unit for severe acute asthma Pediatric Emergency Care 1998 14 104 108 10.1097/00006565-199804000-00004 9583389

[B7] Roberts JS Bratton SL Brogan TV Acute severe asthma: differences in therapies and outcomes among pediatric intensive care units Critical Care Medicine 2002 30 581 585 10.1097/00003246-200203000-00015 11990919

[B8] Dworkin G Kattan M Mechanical ventilation for status asthmaticus in children J Pediatr 1989 114 4 Pt 1 545 549 2494314 10.1016/s0022-3476(89)80691-2

[B9] Mountain RD Heffner JE Brackett NC Sahn SA Acid-base disturbances in acute asthma Chest 1990 98 651 655 10.1378/chest.98.3.651 2118447

[B10] Robertson CF Rubinfeld AR Bowes G Pediatric asthma deaths in Victoria: the mild are at risk Pediatric Pulmonology 1992 13 95 100 10.1002/ppul.1950130207 1495863

[B11] Slater A Shann F Pearson G Paediatric Index of Mortality P. PIM2: a revised version of the Paediatric Index of Mortality Intensive Care Medicine 2003 29 278 285 12541154 10.1007/s00134-002-1601-2

[B12] Mannix R Status asthmaticus in children Current Opin Pediatr 2007 19 281 287 10.1097/MOP.0b013e3280f77531 17505187

[B13] Mannino DM Homa DM Akinbami LJ Moorman JE Gwynn C Redd SC Surveillance for asthma--United States, 1980-1999 Morbidity Mortality Weekly Report 2002 51 1 13 12420904

[B14] Carroll CL Zucker AR The increased cost of complications in children with status asthmaticus Pediatr Pulmonol 2007 42 914 919 10.1002/ppul.20682 17726707

[B15] Eroglu GE Sulun F Status Asthmaticus in Children of Innercity: Findings of a Retrospective Study J Allergy Clin Immunol 2004 113 S308 10.1016/j.jaci.2004.01.601

[B16] Buysse CM de Jongste JC de Hoog M Life-threatening asthma in children: treatment with sodium bicarbonate reduces PCO2 Chest 2005 127 866 870 10.1378/chest.127.3.866 15764769

[B17] Rodrigo GJ Rodrigo CM Status Asthmaticus in Children: Evidence-Based Recommendations Chest 2002 121 667 668 10.1378/chest.121.2.667 11834695

[B18] Werner HA Status Asthmaticus in Children: Evidence-Based Recommendations Chest 2002 121 668 669 10.1378/chest.121.2.667 11834695

[B19] Rachelefsky G Treating exacerbations of asthma in children: the role of systemic corticosteroids Pediatrics 2003 112 382 397 10.1542/peds.112.2.382 12897291

[B20] Papo MC Frank JR A prospective, randomized study of continuous versus intermittent nebulized albuterol for severe status asthmaticus in children Crit Care Medi 1993 21 1479 1486 10.1097/00003246-199310000-00015 8403956

[B21] Bohn DM Intravenous salbutamol in the treatment of status asthmaticus in children Crit Care Med 1984 12 892 896 10.1097/00003246-198410000-00012 6435957

[B22] Carroll CL Schramm CM Noninvasive positive pressure ventilation for the treatment of status asthmaticus in children Ann Allergy Asthma, Immunol 2006 96 454 459 10.1016/S1081-1206(10)60913-1 16597080

[B23] Mehta RM Pearson-Shaver AL Wheeler DS Acute Rhabdomyolysis Complicating Status Asthmaticus in Children: Case Series and Review Pediatr Emerg Care 2006 22 587 591 10.1097/01.pec.0000230711.81646.7a 16912630

[B24] Restrepo RD Peters J Near-fatal asthma: recognition and management Current Opin Pulmon Med 2008 14 13 23 10.1097/MCP.0b013e3282f1982d 18043271

[B25] Ramnath VR Clark S Camargo CA Multicenter study of clinical features of sudden-onset versus slower-onset asthma exacerbations requiring hospitalization Respiratory Care 2007 52 1013 1020 17650357

